# RSAT 2022: regulatory sequence analysis tools

**DOI:** 10.1093/nar/gkac312

**Published:** 2022-05-11

**Authors:** Walter Santana-Garcia, Jaime A Castro-Mondragon, Mónica Padilla-Gálvez, Nga Thi Thuy Nguyen, Ana Elizondo-Salas, Najla Ksouri, François Gerbes, Denis Thieffry, Pierre Vincens, Bruno Contreras-Moreira, Jacques van Helden, Morgane Thomas-Chollier, Alejandra Medina-Rivera

**Affiliations:** Institut de biologie de l’Ecole normale supérieure (IBENS), Ecole normale supérieure, CNRS, INSERM, PSL Université Paris, 75005 Paris, France; Centre for Molecular Medicine Norway (NCMM), Nordic EMBL Partnership, University of Oslo, 0318 Oslo, Norway; Laboratorio Internacional de Investigación sobre el Genoma Humano, Universidad Nacional Autónoma de México, Campus Juriquilla, Blvd Juriquilla 3001, 76230 Santiago de Querétaro, México; Institut de biologie de l’Ecole normale supérieure (IBENS), Ecole normale supérieure, CNRS, INSERM, PSL Université Paris, 75005 Paris, France; Laboratorio Internacional de Investigación sobre el Genoma Humano, Universidad Nacional Autónoma de México, Campus Juriquilla, Blvd Juriquilla 3001, 76230 Santiago de Querétaro, México; Estación Experimental de Aula Dei-CSIC, 50059 Zaragoza, Spain; CNRS, Institut Français de Bioinformatique, IFB-core, UMS 3601, Evry, France; Institut de biologie de l’Ecole normale supérieure (IBENS), Ecole normale supérieure, CNRS, INSERM, PSL Université Paris, 75005 Paris, France; Institut de biologie de l’Ecole normale supérieure (IBENS), Ecole normale supérieure, CNRS, INSERM, PSL Université Paris, 75005 Paris, France; Estación Experimental de Aula Dei-CSIC, 50059 Zaragoza, Spain; CNRS, Institut Français de Bioinformatique, IFB-core, UMS 3601, Evry, France; Aix-Marseille Univ, INSERM UMR_S 1090, Lab Theory and Approaches of Genome Complexity (TAGC), F-13288 Marseille, France; Institut de biologie de l’Ecole normale supérieure (IBENS), Ecole normale supérieure, CNRS, INSERM, PSL Université Paris, 75005 Paris, France; Laboratorio Internacional de Investigación sobre el Genoma Humano, Universidad Nacional Autónoma de México, Campus Juriquilla, Blvd Juriquilla 3001, 76230 Santiago de Querétaro, México

## Abstract

RSAT (Regulatory Sequence Analysis Tools) enables the detection and the analysis of *cis*-regulatory elements in genomic sequences. This software suite performs (i) *de novo* motif discovery (including from genome-wide datasets like ChIP-seq/ATAC-seq) (ii) genomic sequences scanning with known motifs, (iii) motif analysis (quality assessment, comparisons and clustering), (iv) analysis of regulatory variations and (v) comparative genomics. RSAT comprises 50 tools. Six public Web servers (including a teaching server) are offered to meet the needs of different biological communities. RSAT philosophy and originality are: (i) a multi-modal access depending on the user needs, through web forms, command-line for local installation and programmatic web services, (ii) a support for virtually any genome (animals, bacteria, plants, totalizing over 10 000 genomes directly accessible). Since the 2018 NAR Web Software Issue, we have developed a large REST API, extended the support for additional genomes and external motif collections, enhanced some tools and Web forms, and developed a novel tool that builds or refine gene regulatory networks using motif scanning (network-interactions). The RSAT website provides extensive documentation, tutorials and published protocols. RSAT code is under open-source license and now hosted in GitHub. RSAT is available at http://www.rsat.eu/.

## Graphical Abstract

**Graphical abstract:**
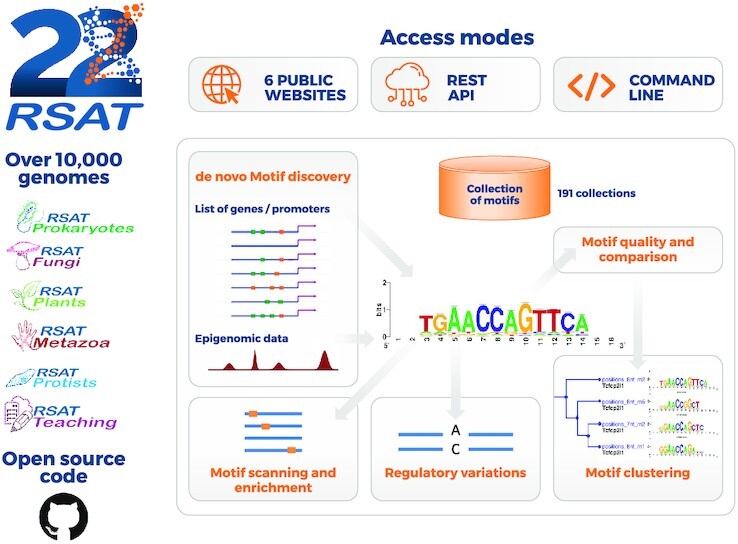
RSAT is an open-source and long-standing suite that enables the detection and the analysis of *cis*-regulatory elements in genomic sequences, for any genomes.

## INTRODUCTION

The Regulatory Sequence Analysis Tools (RSAT) provides a wide range of bioinformatics programs enabling the analysis of genomic regulatory sequences in physiological and disease contexts. RSAT enables users to obtain genomic sequences and perform typical analyses, such as *de novo* motif discovery, or motif scanning to predict transcription factor (TF) binding sites (TFBSs). RSAT functionalities also include original analyses, such as motif quality evaluation, motif comparisons and clustering, detection and analysis of regulatory variants, building of control datasets and comparative genomics to discover motifs based on cross-species conservation. Altogether, the RSAT Web site gives access to 50 tools that can be used individually, or sequentially to perform more complex analyses. RSAT has been well-established since its initial development in 1998 (1,2). It has been regularly updated and extended with novel developments stimulated by advances in the field of regulatory genomics. We summarize here the main functionalities, and describe novelties since the previous NAR Web server issues (3–7).

## RSAT FUNCTIONALITIES

**Figure 1.**
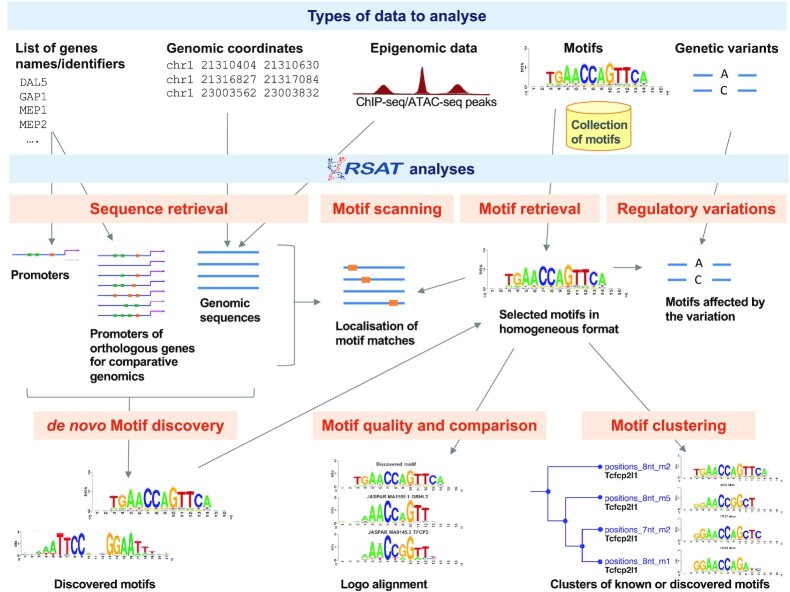
Overview of the main applications of RSAT, with associated input data types.

RSAT tools have been individually described in the previous 2018 NAR update (3), with a historical perspective, as well as by applications (4). We summarize below the main functionalities ordered by data types to analyze, as a useful starting point for novice users (Figure 1). Pointers to the three use cases that exemplify how to combine the tools into routine analysis (3) are indicated.

### Epigenomics datasets such as ChIP-seq or ATAC-seq peaks

Genome-wide datasets obtained from epigenomics experiments (e.g. ChIP-seq, ATAC-seq, ChIP-exo, DNaseI, Cut&Run, Cut&Tag) consists of genomic regions—known as peaks—that are likely bound by a given transcription factor (TF), or associated with open chromatin. The prevalent question is ‘Which TF binding motifs can be detected in the peaks?’

**Figure 2.**
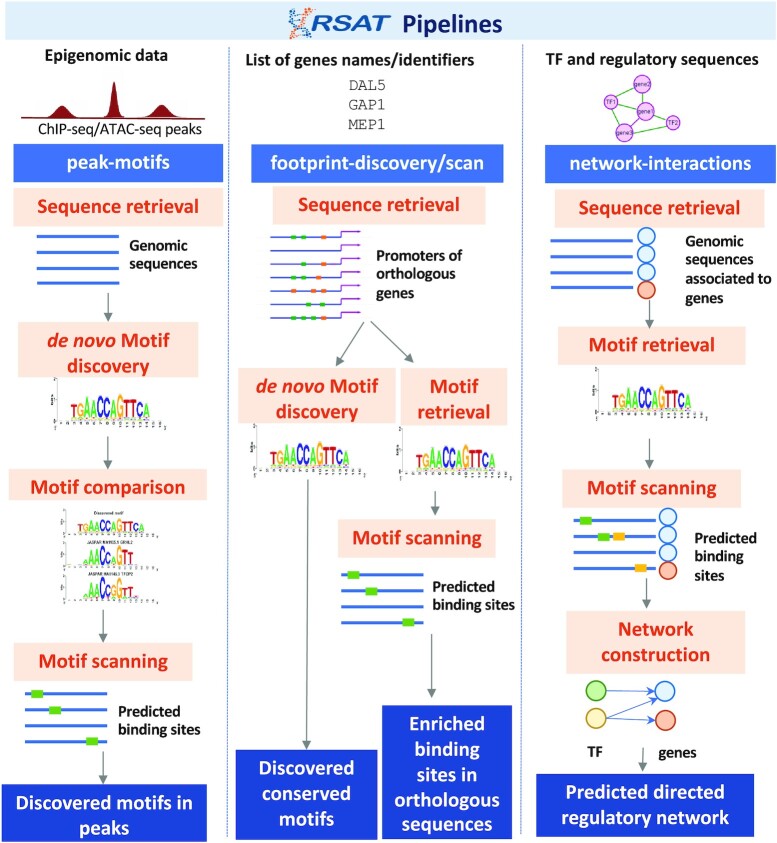
Three pipelines offering pre-defined combinations of RSAT tools (*peak-motifs*, *footprint-scan* and *footprint-discovery*, *network-interactions*).

The peaks can be analyzed with the user-friendly pipeline *peak-motifs* (5,8,9), which relies on *de novo* motif discovery to detect exceptional motifs in a set of sequences. *peak-motifs* runs multiple complementary algorithms [*oligo-analysis* (1), *dyad-analysis* (10), *position-analysis* (11) and *local-word-analysis* (8) that can all be used as independent tools], then compares the predicted motifs with annotated motif databases (*compare-matrices*), and finally predicts the positions of the putative transcription factor binding sites (TFBSs) within the peaks (*matrix-scan* (12) (Figure 2). Two datasets can be provided as input to enable differential analysis.

Alternatively, the peaks can be directly scanned with motifs (e.g. the discovered motifs, or from motif databases such as JASPAR (cf. ‘Motifs represented as Position-Scoring Specific Matrices (PSSM) or consensus sequences’)) to locate putative TFBSs (*dna-pattern* or *matrix-scan* (12)) or to predict potential cis-regulatory modules (*crer-scan* (3)). The tool *matrix-quality* can measure the enrichment of a specific motif within one or more peak datasets (13).

As input peaks must be provided as FASTA-formatted sequences, RSAT provides two tools to extract sequences from genome-wide peak datasets specified in BED-formated genomic coordinates (cf. ‘Genomic coordinates as a BED file’).

Control datasets can be built by selecting sequences at random positions from a given genome (*random-genome-fragments*), or by generating simulated sequences matching the size and composition of the peaks (*random-sequences*).

### Lists of gene names or identifiers

Genome-wide datasets from transcriptomics experiments (e.g. microarrays, RNA-seq), as well as more targeted *in situ* hybridization experiments, typically results in a list of co-expressed genes. A frequent question is ‘Which TFs may co-regulate the expression of these genes?’ The typical analysis workflow consists in (i) retrieving sequences relative to these genes (e.g. promoter) and (ii) performing *de novo* motif discovery or motif scanning (cf. ‘Epigenomics datasets such as ChIP-seq or ATAC-seq peaks’). Given a list of gene names or identifiers, *retrieve-sequences* extracts promoter sequences of locally-installed genomes, while *retrieve-ensembl-seq* (14) retrieves sequences of promoters or other specified features on-the-fly from Ensembl.

To support comparative genomics analyses, *retrieve-ensembl-seq* can also retrieve sequences from homologous genes. On the Plant server, the tool *get-orthologs-compara* additionally returns detailed information on homologous genes in a set of reference organisms, using precomputed Ensembl Compara data (15,16). On the Fungi and Prokaryotes servers, lists of orthologous genes can be obtained with *get-orthologs*. For the subsequent motif analysis step on these servers, *footprint-discovery* (17,18) and *footprint-scan* directly use cross-species conservation to detect putative regulatory signals in non-coding sequences (phylogenetic footprinting) (Figure 2).

Control datasets can be built by randomly selecting genes within a given genome with *random-gene-selection*. Use case 1 (3) combines *get-orthologs-compara, retrieve-sequences* and *matrix-scan* to predict TFBSs of VRN1 within the promoters of the FT1 gene in several plant genomes.

### Motifs represented as Position-Scoring Specific Matrices (PSSM) or consensus sequences

Motifs represented as PSSMs or as consensus sequences may be obtained by *de novo* motif analysis, from databases such as JASPAR (19), or directly from the literature. Some typical questions are (i) ‘Is the motif of good quality ?’, (ii) ‘ Which sequences contain TFBS matching this motif ?’, (iii) ‘Does this motif resemble other motifs ?’.

First, *matrix-quality* (13) aims at assessing the quality of a PSSM on sequence datasets provided by the user, by comparing theoretical and empirical score distributions. Second, *matrix-scan* takes as input motifs to locate putative TFBSs in user-provided sequences (cf. ‘Epigenomics datasets such as ChIP-seq or ATAC-seq peaks’). Third, *compare-matrices* compares two collections of matrices and returns various similarity statistics along with a PSSMs multi-pairwise alignment. *matrix-clustering* (20) regroups similar PSSMs into clusters, builds consensus PSSMs for each cluster and offers a dynamic visualization of aligned PSSMs. We applied *matrix-clustering* to regroup redundant matrices within and across motifs databases, in order to build the RSAT non-redundant motif collections for insects, plants and vertebrates (20). These collections are accessible with *retrieve-matrix* (3), which conveniently offers additional access to 187 external motifs collections, totalizing 454 524 motifs, all homogenized in TRANSFAC format (Supplementary Table S1). These collections include large databases such as JASPAR (19) and FootprintDB (21), as well as more specific ones such as ANISEED (22), RegulonDB (23) or RNA binding motifs, covering all kingdoms (Metazoa, Prokaryotes, Fungi, Plants). JASPAR (19) provides matrix-clustering results for each release, to provide information on the redundancy of motifs (https://jaspar.genereg.net/matrix-clusters/).

As there is no standard format for the PSSMs files, the tool *convert-matrix* performs interconversion between multiple motifs formats, and generates graphical representations of motifs in the form of logos. This allows users to focus on their scientific questions rather than formatting issues.

Control datasets can be built by generating permuted versions of PSSMs with *permute-matrix* or simulated matrix with *random-motif*.

### Genomic coordinates as a BED file

Lists of features (e.g. peaks, predicted TFBSs) with their genomic coordinates are conventionally encoded in BED-formatted files (or GFF/GTF). The usual question is ‘How to identify TFBSs within these regions?’ The first step is to extract the corresponding genomic sequences; we provide user friendly tools with web interfaces to facilitate this task. Sequences can be automatically extracted from the UCSC genome browser with *fetch-sequences-from-UCSC* (3) or from locally -installed genomes with *sequences-from-BED/GFF/VCF*, which internally uses BEDTools and supports repeat-masking (24). Use case 2 (3) combines *retrieve-matrix*, *matrix-clustering*, *sequences-from-BED/GFF/VCF* and *matrix-scan* to generate a non-redundant AP1 motif from multiple annotated motifs, and predict TFBSs of AP1 within ChIP-seq peaks.

### Lists of genetic variants as VCF files

Lists of genetic variants (SNPs, indels) can be retrieved from Genome-wide Association Studies (GWAS) and from databases such as Ensembl. A standard question is ‘Which non-coding variants are affecting TF binding on cis-regulatory elements?’ RSAT provides *variation-tools* (25), a series of programs to obtain information on individual variants, extract their flanking sequences, scan these flanking sequences with motif collections and predict which variants may affect TF binding.

Control datasets can be built by generating permuted versions of PSSMs with *permute-matrix*. Use case 3 (3) combines *convert-variations, retrieve-variation-seq* and *variation-scan* on a VCF-formatted file specifying allelic variants detected in melanoma. It illustrates how scanning the surrounding sequences of the variants with the AP1 motif enables the identification of potential regulatory variants affecting AP1 binding.

## RSAT 2022 NOVELTIES

### RSAT locally installed organisms and motif collections

Since the last NAR Web server issue, we have further extended the number of supported organisms on the public servers, notably for Plants (+25 genomes) and Prokaryotes (+195 genomes). Some organisms were installed upon user request. As of February 2022, RSAT public servers support 10 076 locally installed genomes, including 9 646 Prokaryotes, 245 Fungi, 186 Protists, 91 Metazoa and 93 Plants. Besides, we have extended the number of external motif databases directly accessible in the common TRANSFAC format, from 50 to 187 external databases (cf. ‘Motifs represented as Position-Scoring Specific Matrices (PSSM) or consensus sequences’) (Supplementary Table S1). Some motif collections were added upon user request. Adding new collections can now be made directly by a pull request on GitHub. All collections are freely downloadable to be used independently of RSAT (https://github.com/rsa-tools/motif_databases).

Users genome installation requests for servers are welcomed. In order to get a genome installed users have to contact the RSAT team through email ‘rsat-contact@list01.biologie.ens.fr’ with the information of the requested genome: organism name, genome version, source (*i.e*. NCBI, ENSEMBL) and url link to the genome data. In the case of motif collections, users can also request additions by providing: name, data, URL link and version information.

Furthermore, interested users can install genomes locally in their own RSAT instances. The documentation at https://rsa-tools.github.io/managing-RSAT contains detailed manuals to install genomes from different sources, such as RSAT servers, Ensembl, NCBI and from original FASTA and GTF data files.

### Programmatic REST API access

Our programmatic SOAP/WSDL access is being replaced by the increasingly popular Web service REST API. It provides access to a large set of 49 tools of the RSAT suite. The REST API has been developed with the flask library; its documentation is generated with Swagger UI. Example clients in Python have been written to further help users using this API.

### Updated web interface and tools

Some tools are highly parameterisable, thereby complexifying the corresponding Web forms. We have started to redesign these forms to simplify usage: we are now better separating the mandatory inputs/parameters from the optional ones (see *retrieve-sequence*, *matrix-clustering* and *network-interactions*). Several tools have been updated with additional functionalities or increased efficiency. This is the case of *variation-tools* (cf. ‘Lists of genetic variants as VCF files’), for which haplotype scanning has been improved to assess the regulatory effect in TFBSs of haplotypes with large number of variants (SNPs and indels) in Metazoa and Plants.

### Prediction of TF-gene interactions to build and refine gene regulatory networks

Many efforts have been made to infer gene regulatory networks (GRN) from transcriptomic data, with approaches based on coexpression, orthology or sequence motifs (26), but there is no consensus on a single best method. To further improve the inferred GRNs, it is common to apply motif scanning (pattern-matching) as a second step upon inferred interactions. We introduce *network-interactions*, a new user-friendly GRN reconstruction pipeline based on pattern-matching, which can help refine GRNs generated by other tools (Figure 2). It takes as input two lists: (i) the TFs of interests specified as a list of TF names and (ii) a list of genomic regions associated with gene names (typically promoter/enhancer regions of genes) provided as BED coordinates. A seed network, previously generated from other tools (i.e. based on co-expression), can optionally be provided. *network-interactions* runs *matrix-scan* using one of the motif collections available in RSAT (default is JASPAR’s 2022 vertebrates motif collection (19)) to predict TF-gene interactions. *network-interactions* thereby generates several networks: (i) a complete network for all TF-gene interactions, (ii) another network focusing on TF-TF interactions, (iii) one with 3-step TFs indirect interactions (TF-TF-gene) and (iv) when provided with an input GRN, the overlap and the complements between the input network and the network generated by *network-interactions*, where the overlap includes the putative TF binding information. This novel tool extends RSAT’s suite and offers a straightforward and flexible method to expand and refine GRNs.

### RSAT source code on GitHub and Docker container

The RSAT source code, under AGPL-3.0 open-source license, has been transferred to GitHub, to stimulate community-wise participation in its development: https://github.com/rsa-tools. Additional RSAT documentation is available there as well. A Docker container has been built to analyze the promoters of coexpressed genes in plants (27): https://github.com/eead-csic-compbio/coexpression_motif_discovery.

### Learning to use RSAT

In addition to the above-mentioned use cases, RSAT provides extensive documentation, tutorials and published protocols (4). To target non-expert users, including biologists and biomedical practitioners, the main tools are accessible through web forms with DEMO buttons and tutorials. The latest protocols (28,29) and application (27) focuses on motif discovery in plant genomes; the described approaches can generally be applied to other organisms. Most of our previously published protocols (9,12,30) are still relevant to learn about the underlying algorithms, choosing the relevant parameters and interpreting the results, despite updates in the Web interfaces. Users may also contact us via email or via our Twitter account @RSATools.

## CONCLUSIONS

Compared to alternative programs, RSAT is unique for its wide range of functionalities, extensive motifs collections and >10 000 supported organisms from all kingdoms. The main alternatives are the MEME suite (31), which mainly focuses on motif analyses, and HOMER (32), which primarily focuses on motif discovery. Deep-learning methods are more focused in discovering context-specific TFBS, whereas RSAT aims at providing a complete environment for motif analysis. We aim for RSAT to be usable in combination with other programs (including MEME and HOMER); RSAT thus offers several file format conversion utility tools (*convert-matrix*, *convert-background-models*, *convert-features*, …). After 20 years of existence, RSAT remains one of the most used tools in regulatory genomics. Looking forward, we aim at (i) continuing to enhance the suite in particular to cope with the challenges posed by single cell technologies in terms of data analysis efficiency, and (ii) continuing to ensure long-term maintenance, with packaging in conda, a non-plant docker container and continuous integration on GitHub.

## DATA AVAILABILITY

RSAT public servers are accessible from the RSAT portal at http://www.rsat.eu/. RSAT Web servers can be freely accessed by all users without login requirement. For bioinformatician users, RSAT is accessible (i) as a command-line suite for installation on a local server or on a computer cloud, from its source code https://github.com/rsa-tools, or (ii) via the REST API web programmatic access. RSAT is part of the Service Delivery Plan of the Elixir-France node (European distributed infrastructure for life-science information): https://elixir-europe.org/services/list?field_scientific_domain_tid=All&field_elixir_badge_tid=All&field_type_of_service_tid=All&field_elixir_node_target_id=981&combine=.

RSAT code and documentation is available through GitHub https://github.com/rsa-tools. The Docker container for plants is located at: https://github.com/eead-csic-compbio/coexpression_motif_discovery. Motif collections can be found at https://github.com/rsa-tools/motif_databases.

## Supplementary Material

gkac312_Supplemental_File
