# Supplementary material for: RSAT 2022: regulatory sequence analysis tools
Source: Nucleic Acids Res. 2022 May 11;50(W1):W670–6. doi: 10.1093/nar/gkac312 (PMC9252783; doi:10.1093/nar/gkac312)
Supplement: gkac312_Supplemental_File [file gkac312_supplemental_file.pdf]

Supplementary Table 1 : List of the 187 motif collections integrated in RSAT. This table lists the collection names, date of integration or version, a brief description, and the URL indicating the origin of each database.

Collection name	Database name	Number of motifs	Version	Category	Brief description	URL of origin of the data
Yeastract	Yeastract	732	20130918	Fungi	Yeast motifs compiled from literature and databases	http://www.yeastract.com/
cisBP_S.cerevisiae	cisBP	776	2015-06_v1.02	Fungi	CisBP collects data from >25 sources, including other database such as Transfac, JASPAR, HOCOMOCO, FactorBook, UniProbe, Fly Factor Survey, and dozens of additional publications.	http://cisbp.ccbr.utoronto.ca/
jaspar_core_nonredundant_fungi	Jaspar	183	2020	Fungi	Curated, non-redundant set of profiles, derived from published collections of experimentally defined transcription factor binding sites for eukaryotes.	http://jaspar2020.genereg.net/download/CORE/JASPAR2020_CORE_fungi_non-redundant_pfms_transfac.txt
jaspar_core_redundant_fungi	Jaspar	184	2020	Fungi	Curated set of profiles, derived from published collections of experimentally defined transcription factor binding sites for eukaryotes.	http://jaspar2020.genereg.net/download/CORE/JASPAR2020_CORE_fungi_redundant_pfms_transfac.txt
jaspar_core_+_unvalidated_fungi	Jaspar	231	2022	Fungi	Curated set of profiles, derived from published collections of experimentally defined transcription factor binding sites for eukaryotes.	https://jaspar2022.genereg.net/downloads/
cisBP_Saccharomyces_cerevisiae	cisBP	1288	2019-06_v2.00	Fungi	cisBP specific collection for Saccharomyces_cerevisiae	http://cisbp.ccbr.utoronto.ca/bulk.php
footprintDB	footprintDB	11491	2020-01	Multi-categories	Metadatabase of curated motifs from public databases and the literature	http://floresta.eead.csic.es/footprintdb
jaspar_core_nonredundant_all	Jaspar	1646	2020	Multi-categories	Curated, non-redundant set of profiles, derived from published collections of experimentally defined transcription factor binding sites for eukaryotes.	http://jaspar2020.genereg.net/download/CORE/JASPAR2020_CORE_non-redundant_pfms_transfac.txt
jaspar_core_redundant_all	Jaspar	1964	2020	Multi-categories	Curated set of profiles, derived from published collections of experimentally defined transcription factor binding sites for eukaryotes.	http://jaspar2020.genereg.net/download/CORE/JASPAR2020_CORE_redundant_pfms_transfac.txt
jaspar_unvalidated	Jaspar	337	2020	Multi-categories	JASPAR unvalidated	http://jaspar2020.genereg.net/download/collections/JASPAR2020_UNVALIDATED_pfms_transfac.txt
RSAT_nonredundant_insects_plants_vertebrates	RSAT	2889	2017	Multi-categories	Non-redundant collection obtained by automatic clustering of many external databases with RSAT matrix-clustering	http://pedagogix-tagc.univ-mrs.fr/rsat/data/published_data/Castro_2016_matrix-clustering/
DrosophilaTFs	DrosophilaTFs	61	2015-11	Non-vertebrate Metazoa	Curated motifs derived from two different sources: 1) in vitro binding site selection experiments (e.g. SELEX-like methods), and 2) consensus sequences derived from compiled genomic binding site sequences.	http://bergmanlab.genetics.uga.edu/?page_id=274
FlyFactorSurvey	FlyFactorSurvey	652	2016_06	Non-vertebrate Metazoa	DNA binding specificity data from one-hybrid and DNase I or SELEX methods.	http://mccb.umassmed.edu/ffs/
DMMPMM_drosophila	DMMPMM_drosophila	41	2010_11	Non-vertebrate Metazoa	Motif predictions within DNase I footprinting data	http://autosome.ru/DMMPMM/
IDMMPMM_drosophila	DMMPMM_drosophila	39	2010_11	Non-vertebrate Metazoa	Drosophila transcription factor motifs built from different available experimental sources	http://autosome.ru/iDMMPMM/
Aniseed	Aniseed	275	2020-08	Non-vertebrate Metazoa	Aniseed (Ciona robusta motifs detected by SELEX)	https://www.aniseed.cnrs.fr/aniseed/download/download_data
cisBP_drosophila	cisBP	1427	2014-10_v0.9	Non-vertebrate Metazoa	cisBP drosophila	http://cisbp.ccbr.utoronto.ca/
cisBP_C.elegans	cisBP	1499	2015-05_v1.02	Non-vertebrate Metazoa	cisBP c_elegans	http://cisbp.ccbr.utoronto.ca/
footprintDB-metazoa	footprintDB	7763	2020-01	Non-vertebrate Metazoa	Metadatabase of curated motifs from public databases and the literature [only metazoa DNA motifs]	http://floresta.eead.csic.es/footprintdb
jaspar_core_nonredundant_insects	Jaspar	143	2020	Non-vertebrate Metazoa	Curated, non-redundant set of profiles, derived from published collections of experimentally defined transcription factor binding sites for eukaryotes.	http://jaspar2020.genereg.net/download/CORE/JASPAR2020_CORE_insects_non-redundant_pfms_transfac.txt
jaspar_core_+_unvalidated_insects	Jaspar	156	2022	Non-vertebrate Metazoa	Curated, non-redundant set of profiles, derived from published collections of experimentally defined transcription factor binding sites for eukaryotes.	https://jaspar2022.genereg.net/downloads/
jaspar_core_nonredundant_nematodes	Jaspar	43	2020	Non-vertebrate Metazoa	Curated, non-redundant set of profiles, derived from published collections of experimentally defined transcription factor binding sites for eukaryotes.	http://jaspar2020.genereg.net/download/CORE/JASPAR2020_CORE_nematodes_non-redundant_pfms_transfac.txt
jaspar_core_+_unvalidated_nematodes	Jaspar	45	2022	Non-vertebrate Metazoa	Curated, non-redundant set of profiles, derived from published collections of experimentally defined transcription factor binding sites for eukaryotes.	https://jaspar2022.genereg.net/downloads/
jaspar_core_nonredundant_urochordates	Jaspar	1	2020	Non-vertebrate Metazoa	Curated, non-redundant set of profiles, derived from published collections of experimentally defined transcription factor binding sites for eukaryotes.	http://jaspar2020.genereg.net/download/CORE/JASPAR2020_CORE_urochordates_non-redundant_pfms_transfac.txt
jaspar_core_+_unvalidated_urochordates	Jaspar	132	2022	Non-vertebrate Metazoa	Curated, non-redundant set of profiles, derived from published collections of experimentally defined transcription factor binding sites for eukaryotes.	https://jaspar2022.genereg.net/downloads/
jaspar_core_redundant_insects	Jaspar	153	2020	Non-vertebrate Metazoa	Curated set of profiles, derived from published collections of experimentally defined transcription factor binding sites for eukaryotes.	http://jaspar2020.genereg.net/download/CORE/JASPAR2020_CORE_insects_redundant_pfms_transfac.txt
jaspar_core_redundant_nematodes	Jaspar	43	2020	Non-vertebrate Metazoa	Curated set of profiles, derived from published collections of experimentally defined transcription factor binding sites for eukaryotes.	http://jaspar2020.genereg.net/download/CORE/JASPAR2020_CORE_nematodes_redundant_pfms_transfac.txt

Supplementary Table 1 : List of the 187 motif collections integrated in RSAT. This table lists the collection names, date of integration or version, a brief description, and the URL indicating the origin of each database.						
**Collection name**	**Database name**	**Number of motifs**	**Version**	**Category**	**Brief description**	**URL of origin of the data**
jaspar_core_redundant_urochordates	Jaspar	1	2020	Non-vertebrate Metazoa	Curated set of profiles, derived from published collections of experimentally defined transcription factor binding sites for eukaryotes.	http://jaspar2020.genereg.net/download/CORE/JASPAR2020_CORE_urochordates_redundant_pfms_transfac.txt
RSAT_nonredundant_insects	RSAT	350	2017	Non-vertebrate Metazoa	Non-redundant collection obtained by automatic clustering of many external databases with RSAT matrix-clustering	http://pedagogix-tagc.univ-mrs.fr/rsat/data/published_data/Castro_2016_matrix-clustering/
cisBP_Acyrthosiphon_pisum	cisBP	4022	2019-06_v2.00	Non-vertebrate Metazoa	cisBP Acyrthosiphon_pisum	http://cisbp.ccbr.utoronto.ca/bulk.php
cisBP_Aedes_aegypti	cisBP	3985	2019-06_v2.00	Non-vertebrate Metazoa	cisBP Aedes_aegypti	http://cisbp.ccbr.utoronto.ca/bulk.php
cisBP_Amphimedon_queenslandica	cisBP	2325	2019-06_v2.00	Non-vertebrate Metazoa	cisBP Amphimedon_queenslandica	http://cisbp.ccbr.utoronto.ca/bulk.php
cisBP_Anopheles_darlingi	cisBP	3542	2019-06_v2.00	Non-vertebrate Metazoa	cisBP Anopheles_darlingi	http://cisbp.ccbr.utoronto.ca/bulk.php
cisBP_Anopheles_gambiae	cisBP	4089	2019-06_v2.00	Non-vertebrate Metazoa	cisBP Anopheles_gambiae	http://cisbp.ccbr.utoronto.ca/bulk.php
cisBP_Apis_mellifera	cisBP	4133	2019-06_v2.00	Non-vertebrate Metazoa	cisBP Apis_mellifera	http://cisbp.ccbr.utoronto.ca/bulk.php
cisBP_Atta_cephalotes	cisBP	3404	2019-06_v2.00	Non-vertebrate Metazoa	cisBP Atta_cephalotes	http://cisbp.ccbr.utoronto.ca/bulk.php
cisBP_Bombyx_mori	cisBP	3662	2019-06_v2.00	Non-vertebrate Metazoa	cisBP Bombyx_mori	http://cisbp.ccbr.utoronto.ca/bulk.php
cisBP_Brugia_malayi	cisBP	3105	2019-06_v2.00	Non-vertebrate Metazoa	cisBP Brugia_malayi	http://cisbp.ccbr.utoronto.ca/bulk.php
cisBP_Caenorhabditis_brenneri	cisBP	3169	2019-06_v2.00	Non-vertebrate Metazoa	cisBP Caenorhabditis_brenneri	http://cisbp.ccbr.utoronto.ca/bulk.php
cisBP_Caenorhabditis_briggsae	cisBP	3261	2019-06_v2.00	Non-vertebrate Metazoa	cisBP Caenorhabditis_briggsae	http://cisbp.ccbr.utoronto.ca/bulk.php
cisBP_Caenorhabditis_elegans	cisBP	3438	2019-06_v2.00	Non-vertebrate Metazoa	cisBP Caenorhabditis_elegans	http://cisbp.ccbr.utoronto.ca/bulk.php
cisBP_Caenorhabditis_japonica	cisBP	3107	2019-06_v2.00	Non-vertebrate Metazoa	cisBP Caenorhabditis_japonica	http://cisbp.ccbr.utoronto.ca/bulk.php
cisBP_Caenorhabditis_remanei	cisBP	3289	2019-06_v2.00	Non-vertebrate Metazoa	cisBP Caenorhabditis_remanei	http://cisbp.ccbr.utoronto.ca/bulk.php
cisBP_Ciona_intestinalis	cisBP	3788	2019-06_v2.00	Non-vertebrate Metazoa	cisBP Ciona_intestinalis	http://cisbp.ccbr.utoronto.ca/bulk.php
cisBP_Crassostrea_gigas	cisBP	4030	2019-06_v2.00	Non-vertebrate Metazoa	cisBP Crassostrea_gigas	http://cisbp.ccbr.utoronto.ca/bulk.php
cisBP_Culex_quinquefasciatus	cisBP	3982	2019-06_v2.00	Non-vertebrate Metazoa	cisBP Culex_quinquefasciatus	http://cisbp.ccbr.utoronto.ca/bulk.php
cisBP_Danaus_plexippus	cisBP	3886	2019-06_v2.00	Non-vertebrate Metazoa	cisBP Danaus_plexippus	http://cisbp.ccbr.utoronto.ca/bulk.php
cisBP_Daphnia_pulex	cisBP	678	2019-06_v2.00	Non-vertebrate Metazoa	cisBP Daphnia_pulex	http://cisbp.ccbr.utoronto.ca/bulk.php
cisBP_Dendroctonus_ponderosae	cisBP	3846	2019-06_v2.00	Non-vertebrate Metazoa	cisBP Dendroctonus_ponderosae	http://cisbp.ccbr.utoronto.ca/bulk.php
cisBP_Drosophila_ananassae	cisBP	4154	2019-06_v2.00	Non-vertebrate Metazoa	cisBP Drosophila_ananassae	http://cisbp.ccbr.utoronto.ca/bulk.php
cisBP_Drosophila_erecta	cisBP	4347	2019-06_v2.00	Non-vertebrate Metazoa	cisBP Drosophila_erecta	http://cisbp.ccbr.utoronto.ca/bulk.php
cisBP_Drosophila_grimshawi	cisBP	4226	2019-06_v2.00	Non-vertebrate Metazoa	cisBP Drosophila_grimshawi	http://cisbp.ccbr.utoronto.ca/bulk.php
cisBP_Drosophila_melanogaster	cisBP	4514	2019-06_v2.00	Non-vertebrate Metazoa	cisBP Drosophila_melanogaster	http://cisbp.ccbr.utoronto.ca/bulk.php
cisBP_Drosophila_mojavensis	cisBP	4293	2019-06_v2.00	Non-vertebrate Metazoa	cisBP Drosophila_mojavensis	http://cisbp.ccbr.utoronto.ca/bulk.php
cisBP_Drosophila_persimilis	cisBP	4161	2019-06_v2.00	Non-vertebrate Metazoa	cisBP Drosophila_persimilis	http://cisbp.ccbr.utoronto.ca/bulk.php
cisBP_Drosophila_pseudoobscura	cisBP	4286	2019-06_v2.00	Non-vertebrate Metazoa	cisBP Drosophila_pseudoobscura	http://cisbp.ccbr.utoronto.ca/bulk.php
cisBP_Drosophila_sechellia	cisBP	4352	2019-06_v2.00	Non-vertebrate Metazoa	cisBP Drosophila_sechellia	http://cisbp.ccbr.utoronto.ca/bulk.php
cisBP_Drosophila_simulans	cisBP	4023	2019-06_v2.00	Non-vertebrate Metazoa	cisBP Drosophila_simulans	http://cisbp.ccbr.utoronto.ca/bulk.php
cisBP_Drosophila_virilis	cisBP	4276	2019-06_v2.00	Non-vertebrate Metazoa	cisBP Drosophila_virilis	http://cisbp.ccbr.utoronto.ca/bulk.php
cisBP_Drosophila_willistoni	cisBP	4319	2019-06_v2.00	Non-vertebrate Metazoa	cisBP Drosophila_willistoni	http://cisbp.ccbr.utoronto.ca/bulk.php
cisBP_Drosophila_yakuba	cisBP	4326	2019-06_v2.00	Non-vertebrate Metazoa	cisBP Drosophila_yakuba	http://cisbp.ccbr.utoronto.ca/bulk.php
cisBP_Heliconius_melpomene	cisBP	3647	2019-06_v2.00	Non-vertebrate Metazoa	cisBP Heliconius_melpomene	http://cisbp.ccbr.utoronto.ca/bulk.php
cisBP_Helobdella_robusta	cisBP	3660	2019-06_v2.00	Non-vertebrate Metazoa	cisBP Helobdella_robusta	http://cisbp.ccbr.utoronto.ca/bulk.php
cisBP_Ixodes_scapularis	cisBP	3701	2019-06_v2.00	Non-vertebrate Metazoa	cisBP Ixodes_scapularis	http://cisbp.ccbr.utoronto.ca/bulk.php
cisBP_Loa_loa	cisBP	3085	2019-06_v2.00	Non-vertebrate Metazoa	cisBP Loa_loa	http://cisbp.ccbr.utoronto.ca/bulk.php
cisBP_Lottia_gigantea	cisBP	3989	2019-06_v2.00	Non-vertebrate Metazoa	cisBP Lottia_gigantea	http://cisbp.ccbr.utoronto.ca/bulk.php
cisBP_Lucilia_cuprina	cisBP	3942	2019-06_v2.00	Non-vertebrate Metazoa	cisBP Lucilia_cuprina	http://cisbp.ccbr.utoronto.ca/bulk.php
cisBP_Megaselia_scalaris	cisBP	2683	2019-06_v2.00	Non-vertebrate Metazoa	cisBP Megaselia_scalaris	http://cisbp.ccbr.utoronto.ca/bulk.php
cisBP_Melitaea_cinxia	cisBP	3363	2019-06_v2.00	Non-vertebrate Metazoa	cisBP Melitaea_cinxia	http://cisbp.ccbr.utoronto.ca/bulk.php
cisBP_Nasonia_vitripennis	cisBP	4064	2019-06_v2.00	Non-vertebrate Metazoa	cisBP Nasonia_vitripennis	http://cisbp.ccbr.utoronto.ca/bulk.php
cisBP_Nematostella_vectensis	cisBP	3571	2019-06_v2.00	Non-vertebrate Metazoa	cisBP Nematostella_vectensis	http://cisbp.ccbr.utoronto.ca/bulk.php
cisBP_Octopus_bimaculoides	cisBP	3795	2019-06_v2.00	Non-vertebrate Metazoa	cisBP Octopus_bimaculoides	http://cisbp.ccbr.utoronto.ca/bulk.php
cisBP_Onchocerca_volvulus	cisBP	2988	2019-06_v2.00	Non-vertebrate Metazoa	cisBP Onchocerca_volvulus	http://cisbp.ccbr.utoronto.ca/bulk.php
cisBP_Pediculus_humanus	cisBP	4057	2019-06_v2.00	Non-vertebrate Metazoa	cisBP Pediculus_humanus	http://cisbp.ccbr.utoronto.ca/bulk.php
cisBP_Pristionchus_pacificus	cisBP	2683	2019-06_v2.00	Non-vertebrate Metazoa	cisBP Pristionchus_pacificus	http://cisbp.ccbr.utoronto.ca/bulk.php
cisBP_Rhodnius_prolixus	cisBP	3380	2019-06_v2.00	Non-vertebrate Metazoa	cisBP Rhodnius_prolixus	http://cisbp.ccbr.utoronto.ca/bulk.php
cisBP_Schistosoma_mansoni	cisBP	3181	2019-06_v2.00	Non-vertebrate Metazoa	cisBP Schistosoma_mansoni	http://cisbp.ccbr.utoronto.ca/bulk.php
cisBP_Solenopsis_invicta	cisBP	3658	2019-06_v2.00	Non-vertebrate Metazoa	cisBP Solenopsis_invicta	http://cisbp.ccbr.utoronto.ca/bulk.php
cisBP_Strigamia_maritima	cisBP	3791	2019-06_v2.00	Non-vertebrate Metazoa	cisBP Strigamia_maritima	http://cisbp.ccbr.utoronto.ca/bulk.php

**Supplementary Table 1 : List of the 187 motif collections integrated in RSAT. This table lists the collection names, date of integration or version, a brief description, and the URL indicating the origin of each database.**						
**Collection name**	**Database name**	**Number of motifs**	**Version**	**Category**	**Brief description**	**URL of origin of the data**
cisBP_Strongylocentrotus_purpuratus	cisBP	3989	2019-06_v2.00	Non-vertebrate Metazoa	cisBP Strongylocentrotus_purpuratus	http://cisbp.ccbr.utoronto.ca/bulk.php
cisBP_Tetranychus_urticae	cisBP	3909	2019-06_v2.00	Non-vertebrate Metazoa	cisBP Tetranychus_urticae	http://cisbp.ccbr.utoronto.ca/bulk.php
cisBP_Tribolium_castaneum	cisBP	4101	2019-06_v2.00	Non-vertebrate Metazoa	cisBP Tribolium_castaneum	http://cisbp.ccbr.utoronto.ca/bulk.php
cisBP_Trichinella_spiralis	cisBP	3131	2019-06_v2.00	Non-vertebrate Metazoa	cisBP Trichinella_spiralis	http://cisbp.ccbr.utoronto.ca/bulk.php
cisBP_Trichoplax_adhaerens	cisBP	2901	2019-06_v2.00	Non-vertebrate Metazoa	cisBP Trichoplax_adhaerens	http://cisbp.ccbr.utoronto.ca/bulk.php
cisBP_Zootermopsis_nevadensis	cisBP	3945	2019-06_v2.00	Non-vertebrate Metazoa	cisBP Zootermopsis_nevadensis	http://cisbp.ccbr.utoronto.ca/bulk.php
ArabidopsisPBM	ArabidopsisPBM	108	2015-11	Plants	Motifs derived from protein-binding microarrays (PBMs)	http://www.pnas.org/content/111/6/2367.abstract
Athamap	Athamap	84	2015-11	Plants	Compiled motifs from databases and literature	http://www.athamap.de/
cistrome	cistrome	862	2016-06	Plants	Motifs derived from DNA affinity purification sequencing (DAP-seq), a high-throughput TF binding site discovery method that interrogates genomic DNA with in-vitro-expressed TFs	http://neomorph.salk.edu/dev/pages/shhuang/dap_web/pages/index.php
cisBP_A_thaliana	cisBP	309	2015-06_v1.02	Plants	cisBP a_thaliana	http://cisbp.ccbr.utoronto.ca/
footprintDB-plants	footprintDB	1997	2020-01	Plants	Metadatabase of curated motifs from public databases and the literature [only plant DNA motifs]	http://floresta.eead.csic.es/footprintdb
jaspar_core_nonredundant_plants	Jaspar	530	2020	Plants	Curated, non-redundant set of profiles, derived from published collections of experimentally defined transcription factor binding sites for eukaryotes.	http://jaspar2020.genereg.net/download/CORE/JASPAR2020_CORE_plants_non-redundant_pfms_transfac.txt
jaspar_core_redundant_plants	Jaspar	572	2020	Plants	Curated set of profiles, derived from published collections of experimentally defined transcription factor binding sites for eukaryotes.	http://jaspar2020.genereg.net/download/CORE/JASPAR2020_CORE_plants_redundant_pfms_transfac.txt
jaspar_core_+_unvalidated_plants	Jaspar	769	2022	Plants	Curated, non-redundant set of profiles, derived from published collections of experimentally defined transcription factor binding sites for eukaryotes.	https://jaspar2022.genereg.net/downloads/
RSAT_nonredundant_plants	RSAT	306	2017	Plants	Non-redundant collection obtained by automatic clustering of many external databases with RSAT matrix-clustering	http://pedagogix-tagc.univ-mrs.fr/rsat/data/published_data/Castro_2016_matrix-clustering/
cisBP_Arabidopsis_thaliana	cisBP	1653	2019-09_v2.00	Plants	cisBP Arabidopsis_thaliana	http://cisbp.ccbr.utoronto.ca/bulk.php
cisBP_Aegilops_tauschii	cisBP	1177	2019-06_v2.00	Plants	cisBP Aegilops_tauschii	http://cisbp.ccbr.utoronto.ca/bulk.php
cisBP_Amborella_trichopoda	cisBP	1291	2019-06_v2.00	Plants	cisBP Amborella_trichopoda	http://cisbp.ccbr.utoronto.ca/bulk.php
cisBP_Arabidopsis_lyrata	cisBP	1556	2019-06_v2.00	Plants	cisBP Arabidopsis_lyrata	http://cisbp.ccbr.utoronto.ca/bulk.php
cisBP_Brachypodium_distachyon	cisBP	1295	2019-06_v2.00	Plants	cisBP Brachypodium_distachyon	http://cisbp.ccbr.utoronto.ca/bulk.php
cisBP_Brassica_napus	cisBP	25	2019-06_v2.00	Plants	cisBP Brassica_napus	http://cisbp.ccbr.utoronto.ca/bulk.php
cisBP_Brassica_oleracea	cisBP	1539	2019-06_v2.00	Plants	cisBP Brassica_oleracea	http://cisbp.ccbr.utoronto.ca/bulk.php
cisBP_Brassica_rapa	cisBP	1532	2019-06_v2.00	Plants	cisBP Brassica_rapa	http://cisbp.ccbr.utoronto.ca/bulk.php
cisBP_Chlamydomonas_reinhardtii	cisBP	340	2019-06_v2.00	Plants	cisBP Chlamydomonas_reinhardtii	http://cisbp.ccbr.utoronto.ca/bulk.php
cisBP_Cucumis_sativus	cisBP	1398	2019-06_v2.00	Plants	cisBP Cucumis_sativus	http://cisbp.ccbr.utoronto.ca/bulk.php
cisBP_Fragaria_vesca	cisBP	1363	2019-06_v2.00	Plants	cisBP Fragaria_vesca	http://cisbp.ccbr.utoronto.ca/bulk.php
cisBP_Glycine_max	cisBP	1491	2019-06_v2.00	Plants	cisBP Glycine_max	http://cisbp.ccbr.utoronto.ca/bulk.php
cisBP_Gossypium_raimondii	cisBP	1448	2019-06_v2.00	Plants	cisBP Gossypium_raimondii	http://cisbp.ccbr.utoronto.ca/bulk.php
cisBP_Helianthus_annuus	cisBP	4	2019-06_v2.00	Plants	cisBP Helianthus_annuus	http://cisbp.ccbr.utoronto.ca/bulk.php
cisBP_Hordeum_vulgare	cisBP	1279	2019-06_v2.00	Plants	cisBP Hordeum_vulgare	http://cisbp.ccbr.utoronto.ca/bulk.php
cisBP_Leersia_perrieri	cisBP	1300	2019-06_v2.00	Plants	cisBP Leersia_perrieri	http://cisbp.ccbr.utoronto.ca/bulk.php
cisBP_Manihot_esculenta	cisBP	1425	2019-06_v2.00	Plants	cisBP Manihot_esculenta	http://cisbp.ccbr.utoronto.ca/bulk.php
cisBP_Medicago_truncatula	cisBP	1422	2019-06_v2.00	Plants	cisBP Medicago_truncatula	http://cisbp.ccbr.utoronto.ca/bulk.php
cisBP_Musa_acuminata	cisBP	1391	2019-06_v2.00	Plants	cisBP Musa_acuminata	http://cisbp.ccbr.utoronto.ca/bulk.php
cisBP_Oryza_barthii	cisBP	1296	2019-06_v2.00	Plants	cisBP Oryza_barthii	http://cisbp.ccbr.utoronto.ca/bulk.php
cisBP_Oryza_brachyantha	cisBP	1264	2019-06_v2.00	Plants	cisBP Oryza_brachyantha	http://cisbp.ccbr.utoronto.ca/bulk.php
cisBP_Oryza_glaberrima	cisBP	1281	2019-06_v2.00	Plants	cisBP Oryza_glaberrima	http://cisbp.ccbr.utoronto.ca/bulk.php
cisBP_Oryza_glumaepatula	cisBP	1311	2019-06_v2.00	Plants	cisBP Oryza_glumaepatula	http://cisbp.ccbr.utoronto.ca/bulk.php
cisBP_Oryza_indica	cisBP	1310	2019-06_v2.00	Plants	cisBP Oryza_indica	http://cisbp.ccbr.utoronto.ca/bulk.php
cisBP_Oryza_longistaminata	cisBP	1151	2019-06_v2.00	Plants	cisBP Oryza_longistaminata	http://cisbp.ccbr.utoronto.ca/bulk.php
cisBP_Oryza_meridionalis	cisBP	1289	2019-06_v2.00	Plants	cisBP Oryza_meridionalis	http://cisbp.ccbr.utoronto.ca/bulk.php
cisBP_Oryza_nivara	cisBP	1297	2019-06_v2.00	Plants	cisBP Oryza_nivara	http://cisbp.ccbr.utoronto.ca/bulk.php
cisBP_Oryza_punctata	cisBP	1341	2019-06_v2.00	Plants	cisBP Oryza_punctata	http://cisbp.ccbr.utoronto.ca/bulk.php
cisBP_Oryza_rufipogon	cisBP	1302	2019-06_v2.00	Plants	cisBP Oryza_rufipogon	http://cisbp.ccbr.utoronto.ca/bulk.php
cisBP_Oryza_sativa	cisBP	1321	2019-06_v2.00	Plants	cisBP Oryza_sativa	http://cisbp.ccbr.utoronto.ca/bulk.php
cisBP_Ostreococcus_lucimarinus	cisBP	418	2019-06_v2.00	Plants	cisBP Ostreococcus_lucimarinus	http://cisbp.ccbr.utoronto.ca/bulk.php
cisBP_Phaseolus_vulgaris	cisBP	12	2019-06_v2.00	Plants	cisBP Phaseolus_vulgaris	http://cisbp.ccbr.utoronto.ca/bulk.php

Supplementary Table 1 : List of the 187 motif collections integrated in RSAT. This table lists the collection names, date of integration or version, a brief description, and the URL indicating the origin of each database.

Collection name	Database name	Number of motifs	Version	Category	Brief description	URL of origin of the data
cisBP_Physcomitrella_patens	cisBP	1033	2019-06_v2.00	Plants	cisBP Physcomitrella_patens	http://cisbp.ccbr.utoronto.ca/bulk.php
cisBP_Populus_trichocarpa	cisBP	1428	2019-06_v2.00	Plants	cisBP Populus_trichocarpa	http://cisbp.ccbr.utoronto.ca/bulk.php
cisBP_Prunus_mume	cisBP	1386	2019-06_v2.00	Plants	cisBP Prunus_mume	http://cisbp.ccbr.utoronto.ca/bulk.php
cisBP_Prunus_persica	cisBP	1406	2019-06_v2.00	Plants	cisBP Prunus_persica	http://cisbp.ccbr.utoronto.ca/bulk.php
cisBP_Selaginella_moellendorffii	cisBP	1077	2019-06_v2.00	Plants	cisBP Selaginella_moellendorffii	http://cisbp.ccbr.utoronto.ca/bulk.php
cisBP_Setaria_italica	cisBP	1305	2019-06_v2.00	Plants	cisBP Setaria_italica	http://cisbp.ccbr.utoronto.ca/bulk.php
cisBP_Solanum_lycopersicum	cisBP	1399	2019-06_v2.00	Plants	cisBP Solanum_lycopersicum	http://cisbp.ccbr.utoronto.ca/bulk.php
cisBP_Solanum_tuberosum	cisBP	1361	2019-06_v2.00	Plants	cisBP Solanum_tuberosum	http://cisbp.ccbr.utoronto.ca/bulk.php
cisBP_Sorghum_bicolor	cisBP	1306	2019-06_v2.00	Plants	cisBP Sorghum_bicolor	http://cisbp.ccbr.utoronto.ca/bulk.php
cisBP_Triticum_aestivum	cisBP	1293	2019-06_v2.00	Plants	cisBP Triticum_aestivum	http://cisbp.ccbr.utoronto.ca/bulk.php
cisBP_Triticum_urartu	cisBP	1124	2019-06_v2.00	Plants	cisBP Triticum_urartu	http://cisbp.ccbr.utoronto.ca/bulk.php
cisBP_Vitis_vinifera	cisBP	1418	2019-06_v2.00	Plants	cisBP Vitis_vinifera	http://cisbp.ccbr.utoronto.ca/bulk.php
cisBP_Zea_mays	cisBP	1334	2019-06_v2.00	Plants	cisBP Zea_mays	http://cisbp.ccbr.utoronto.ca/bulk.php
DBTBS	DBTBS	88	2015-11	Prokaryotes	Curated Bacillus subtilis motifs obtained by compiling invididual experimentally-dereived cis-regulatory elements	http://dbtbs.hgc.jp/
regulonDB	regulonDB	92	2015_08	Prokaryotes	Motifs inferred from the curated compilation of binding sites (Escherichia coli K-12)	http://regulondb.ccg.unam.mx/
ATtRACT	ATtRACT	1322	2017	RNA binding	Motifs compiled from hand-curated experimentally validated data from CISBP-RNA, SpliceAid-F, RBPDB, ASD databases, and predicted from Protein-RNA complexes present in Protein Data Bank database through computational analyses.	https://attract.cnic.es/download
RBPDB	RBPDB	71	2017	RNA binding	Collection of experimental observations of RNA-binding sites, both in vitro and in vivo, manually curated from primary literature	http://rbpdb.ccbr.utoronto.ca/downloads/PFMDir.zip
CISBP-RNA	cisBP	11897	2017	RNA binding	Catalog of Inferred Sequence Binding Proteins of RNA (CISBP-RNA) is a library of RNA binding protein (RBP) motifs and specificities.	http://cisbp-rna.ccbr.utoronto.ca/
ENCODE	ENCODE	2065	2018-03	Vertebrates	Motif analysis for 427 human ENCODE ChIP-seq data	https://www.ncbi.nlm.nih.gov/pubmed/24335146
epigram	epigram	589	2016-06	Vertebrates	cis elements represent interactions with the site-specific DNA-binding factors that establish and maintain epigenomic modifications.	http://wanglab.ucsd.edu/star/epigram/
Hocomoco_human	Hocomoco	771	2017-10	Vertebrates	Motifs discovered with the program ChIPMunk on ChIP-seq datasets	http://hocomoco11.autosome.ru/
Hocomoco_mouse	Hocomoco	531	2017-10	Vertebrates	Motifs discovered with the program ChIPMunk on ChIP-seq datasets	http://hocomoco11.autosome.ru/
Homer	Homer	332	2016-07	Vertebrates	Homer (Human TF motifs)	http://homer.salk.edu/homer/motif/motifDatabase.html
hPDI	hPDI	437	2016-06	Vertebrates	hPDI (Human TFs identified by protein microarray assays)	http://bioinfo.wilmer.jhu.edu/PDI/
HT_Methyl_Selex	HT_Selex	923	2018-03	Vertebrates	Binding specificities of full-length transcription factors and extended DNA binding domains to unmethylated and CpG-methylated DNA by using methylation-sensitive SELEX	http://science.sciencemag.org/content/356/6337/eaaj2239.long
HT_Selex	HT_Selex	864	2018-03	Vertebrates	Binding specificities of full-length transcription factors and extended DNA binding domains to unmethylated and CpG-methylated DNA by using methylation-sensitive SELEX	http://science.sciencemag.org/content/356/6337/eaaj2239.long
Jolma_2013	Jolma	818	2015-11	Vertebrates	Sequence-specific binding of human TFs using high-throughput SELEX and ChIP sequencing	http://www.cell.com/abstract/S0092-8674%2812%2901496-1
Human TFs dimers	Jolma	664	2016-05	Vertebrates	Human_TF_dimers (Human TFs dimers) Cooperative binding of multiple TFs: CAP-SELEX analysis of 9,400 TF–TF–DNA interactions	http://www.nature.com/nature/journal/v527/n7578/full/nature15518.html
NCAP-SELEX	CAP-SELEX	195	2018-09	Vertebrates	NCAP-SELEX (HUMAN TFs)	https://doi.org/10.1038/s41586-018-0549-5
CAP-SELEX	CAP-SELEX	186	2018-09	Vertebrates	CAP-SELEX (HUMAN TFs)	https://doi.org/10.1038/s41586-018-0549-5
ZF_ChIP-exo	ZF_ChIP-exo	126	2020-07	Vertebrates	ZF_ChIP-exo (C2H2 zinc fingers)	https://www.biorxiv.org/content/10.1101/630756v4
ZF_ChIP-seq	ZF_ChIP-seq	109	2020-07	Vertebrates	ZF_ChIP-seq (C2H2 zinc fingers)	https://www.biorxiv.org/content/10.1101/630756v4
MethMotif	MethMotif	519	2018-11	Vertebrates	Methylated motifs discovered in 11 cell-types (Human TFs)	https://academic.oup.com/nar/advance-article/doi/10.1093/nar/gky1005/5150233
cisBP_human	cisBP	1832	2014-10_v0.9	Vertebrates	CisBP collects data from >25 sources, including other database such as Transfac, JASPAR, HOCOMOCO, FactorBook, UniProbe, Fly Factor Survey, and dozens of additional publications.	http://cisbp.ccbr.utoronto.ca/
cisBP_mouse	cisBP	1715	2014-10_v0.9	Vertebrates	CisBP collects data from >25 sources, including other database such as Transfac, JASPAR, HOCOMOCO, FactorBook, UniProbe, Fly Factor Survey, and dozens of additional publications.	http://cisbp.ccbr.utoronto.ca/
jaspar_core_nonredundant_vertebrates	Jaspar	746	2020	Vertebrates	JASPAR core nonredundant vertebrates	http://jaspar2020.genereg.net/download/CORE/JASPAR2020_CORE_vertebrates_non-redundant_pfms_transfac.txt
jaspar_core_redundant_vertebrates	Jaspar	1011	2020	Vertebrates	JASPAR core redundant vertebrates	http://jaspar2020.genereg.net/download/CORE/JASPAR2020_CORE_vertebrates_redundant_pfms_transfac.txt
jaspar_core_+_unvalidated_vertebrates	Jaspar	1167	2022	Vertebrates	JASPAR core + unvalidated vertebrates	https://jaspar2022.genereg.net/downloads/
RSAT_nonredundant_vertebrates	RSAT	2233	2017	Vertebrates	RSAT non-redundant vertebrates	http://pedagogix-tagc.univ-mrs.fr/rsat/data/published_data/Castro_2016_matrix-clustering/
cisBP_Ailuropoda_melanoleuca	cisBP	5863	2019-06_v2.00	Vertebrates	cisBP Ailuropoda_melanoleuca	http://cisbp.ccbr.utoronto.ca/bulk.php

Supplementary Table 1 : List of the 187 motif collections integrated in RSAT. This table lists the collection names, date of integration or version, a brief description, and the URL indicating the origin of each database.						
**Collection name**	**Database name**	**Number of motifs**	**Version**	**Category**	**Brief description**	**URL of origin of the data**
cisBP_Anas_platyrhynchos	cisBP	5038	2019-06_v2.00	Vertebrates	cisBP Anas_platyrhynchos	http://cisbp.ccbr.utoronto.ca/bulk.php
cisBP_Astyanax_mexicanus	cisBP	5191	2019-06_v2.00	Vertebrates	cisBP Astyanax_mexicanus	http://cisbp.ccbr.utoronto.ca/bulk.php
cisBP_Bos_taurus	cisBP	5895	2019-06_v2.00	Vertebrates	cisBP Bos_taurus	http://cisbp.ccbr.utoronto.ca/bulk.php
cisBP_Callithrix_jacchus	cisBP	6089	2019-06_v2.00	Vertebrates	cisBP Callithrix_jacchus	http://cisbp.ccbr.utoronto.ca/bulk.php
cisBP_Cavia_porcellus	cisBP	5683	2019-06_v2.00	Vertebrates	cisBP Cavia_porcellus	http://cisbp.ccbr.utoronto.ca/bulk.php
cisBP_Chlorocebus_sabaeus	cisBP	6176	2019-06_v2.00	Vertebrates	cisBP Chlorocebus_sabaeus	http://cisbp.ccbr.utoronto.ca/bulk.php
cisBP_Choloepus_hoffmanni	cisBP	4069	2019-06_v2.00	Vertebrates	cisBP Choloepus_hoffmanni	http://cisbp.ccbr.utoronto.ca/bulk.php
cisBP_Danio_rerio	cisBP	5320	2019-06_v2.00	Vertebrates	cisBP Danio_rerio	http://cisbp.ccbr.utoronto.ca/bulk.php
cisBP_Dasypus_novemcinctus	cisBP	5871	2019-06_v2.00	Vertebrates	cisBP Dasypus_novemcinctus	http://cisbp.ccbr.utoronto.ca/bulk.php
cisBP_Dipodomys_ordii	cisBP	4810	2019-06_v2.00	Vertebrates	cisBP Dipodomys_ordii	http://cisbp.ccbr.utoronto.ca/bulk.php
cisBP_Echinops_telfairi	cisBP	4813	2019-06_v2.00	Vertebrates	cisBP Echinops_telfairi	http://cisbp.ccbr.utoronto.ca/bulk.php
cisBP_Equus_caballus	cisBP	5765	2019-06_v2.00	Vertebrates	cisBP Equus_caballus	http://cisbp.ccbr.utoronto.ca/bulk.php
cisBP_Erinaceus_europaeus	cisBP	4783	2019-06_v2.00	Vertebrates	cisBP Erinaceus_europaeus	http://cisbp.ccbr.utoronto.ca/bulk.php
cisBP_Gallus_gallus	cisBP	5272	2019-06_v2.00	Vertebrates	cisBP Gallus_gallus	http://cisbp.ccbr.utoronto.ca/bulk.php
cisBP_Homo_sapiens	cisBP	6607	2019-06_v2.00	Vertebrates	cisBP Homo_sapiens	http://cisbp.ccbr.utoronto.ca/bulk.php
cisBP_Mus_musculus	cisBP	6014	2019-06_v2.00	Vertebrates	cisBP Mus_musculus	http://cisbp.ccbr.utoronto.ca/bulk.php
cisBP_Oreochromis_niloticus	cisBP	5296	2019-06_v2.00	Vertebrates	cisBP Oreochromis_niloticus	http://cisbp.ccbr.utoronto.ca/bulk.php
cisBP_Rattus_norvegicus	cisBP	5946	2019-06_v2.00	Vertebrates	cisBP Rattus_norvegicus	http://cisbp.ccbr.utoronto.ca/bulk.php
cisBP_Xenopus_tropicalis	cisBP	5393	2019-06_v2.00	Vertebrates	cisBP Xenopus_tropicalis	http://cisbp.ccbr.utoronto.ca/bulk.php
FactorBook_ChIP-seq	FactorBook	6921	2022-04	Vertebrates	Motifs discovered by MEME-ChIP in ENCODE hIP-seq datasets	https://www.factorbook.org/motif/human/download
FactorBook_SELEX	FactorBook	6700	2022-04	Vertebrates	Motifs discovered by ZMotif in reads from SELEX-seq experiments	https://www.factorbook.org/motif/human/download
